# Supplementary material for: Tbx5 drives Aldh1a2 expression to regulate a RA-Hedgehog-Wnt gene regulatory network coordinating cardiopulmonary development
Source: eLife. 2021 Oct 13;10:e69288. doi: 10.7554/eLife.69288 (PMC8555986; doi:10.7554/eLife.69288)
Supplement: Transparent reporting form [file elife-69288-transrepform1.docx]

***eLife’s* transparent reporting form**

We encourage authors to provide detailed information *within their submission* to facilitate the interpretation and replication of experiments. Authors can upload supporting documentation to indicate the use of appropriate reporting guidelines for health-related research (see [EQUATOR Network](http://www.equator-network.org/%20)), life science research (see the [BioSharing Information Resource](https://biosharing.org/)), or the [ARRIVE guidelines](http://www.plosbiology.org/article/info:doi/10.1371/journal.pbio.1000412) for reporting work involving animal research. Where applicable, authors should refer to any relevant reporting standards documents in this form.

If you have any questions, please consult our Journal Policies and/or contact us: [editorial@elifesciences.org](mailto:editorial@elifesciences.org).

**Sample-size estimation**

- You should state whether an appropriate sample size was computed when the study was being designed
- You should state the statistical method of sample size computation and any required assumptions
- If no explicit power analysis was used, you should describe how you decided what sample (replicate) size (number) to use

Please outline where this information can be found within the submission (e.g., sections or figure legends), or explain why this information doesn’t apply to your submission:

- We have stated the sample size in each figure and/or legend, with additional details in the methods.
- We did not perform any power analysis to decide the sample size or replicate number.
- For each experiment we used as many embryos or tissue explants /condition that were practical to obtain from a single group sibling embryos.

**Replicates**

- You should report how often each experiment was performed
- You should include a definition of biological versus technical replication
- The data obtained should be provided and sufficient information should be provided to indicate the number of independent biological and/or technical replicates
- If you encountered any outliers, you should describe how these were handled
- Criteria for exclusion/inclusion of data should be clearly stated
- High-throughput sequence data should be uploaded before submission, with a private link for reviewers provided (these are available from both GEO and ArrayExpress)

Please outline where this information can be found within the submission (e.g., sections or figure legends), or explain why this information doesn’t apply to your submission:

- Each experiment as independently repeated at least three times. Details on the number of biological replicates / experiment and definitions of biological replicates is reported in each figure legend. We did not perform any technical replicates.
- Each RT-PCR and luciferase assays was performed in biological triplicate with 4-5 explants /replicate. Individual data points are shown with the mean and standard deviation.
- For each in situ hybridization and immunostaining experiment the number of embryos with the indicated phenotype or expression pattern is reported.
- We did not encounter any outliers and no data was excluded.
- The new Tbx5 E14.5 lung ChIP-seq data is available at GEO: GSE167207

**Statistical reporting**

- Statistical analysis methods should be described and justified
- Raw data should be presented in figures whenever informative to do so (typically when N per group is less than 10)
- For each experiment, you should identify the statistical tests used, exact values of N, definitions of center, methods of multiple test correction, and dispersion and precision measures (e.g., mean, median, SD, SEM, confidence intervals; and, for the major substantive results, a measure of effect size (e.g., Pearson's r, Cohen's d)
- Report exact p-values wherever possible alongside the summary statistics and 95% confidence intervals. These should be reported for all key questions and not only when the p-value is less than 0.05.

Please outline where this information can be found within the submission (e.g., sections or figure legends), or explain why this information doesn’t apply to your submission:

- The statistical tests are described in the methods and reported in each figure legend.
- Individual data points for each biological replicate from representative experiment (RT-PCR and luciferase) are shown along with mean and standard deviation.
- Where appropriate we have included the exact p-values.

(For large datasets, or papers with a very large number of statistical tests, you may upload a single table file with tests, Ns, etc., with reference to sections in the manuscript.)

**Group allocation**

- Indicate how samples were allocated into experimental groups (in the case of clinical studies, please specify allocation to treatment method); if randomization was used, please also state if restricted randomization was applied
- Indicate if masking was used during group allocation, data collection and/or data analysis

Please outline where this information can be found within the submission (e.g., sections or figure legends), or explain why this information doesn’t apply to your submission:

Samples were not randomized or blinded. Samples were grouped based on the experimental manipulation.

**Additional data files (“source data”)**

- We encourage you to upload relevant additional data files, such as numerical data that are represented as a graph in a figure, or as a summary table
- Where provided, these should be in the most useful format, and they can be uploaded as “Source data” files linked to a main figure or table
- Include model definition files including the full list of parameters used
- Include code used for data analysis (e.g., R, MatLab)
- Avoid stating that data files are “available upon request”

Please indicate the figures or tables for which source data files have been provided:

We include the source date for the following figures in excel spreadsheets

Figure2_supplement1_Source Data 1

Figure4-Source Data 1_Fig4B

Figure4-Source Data 2_Fig4H

Figure4-Source Data 3_Fig4I

Figure4-Source Data 4_Fig4J

Figure5-Source Data 1_Fig5B

Figure5-Source Data 2_Fig5D

Figure5_supplement2_Source Data 1

Figure6-Source Data 1_Fig6B

Figure6-Source Data2_Fig6E

Figure6_supplement2_Source Data 1
